# Diet Diversity and Feeding Practices in Toddlers with and Without Food Allergy—A Cross-Sectional Study

**DOI:** 10.3390/nu17203212

**Published:** 2025-10-13

**Authors:** Agata Stróżyk, Andrea Horvath, Elżbieta Jarocka-Cyrta, Daria Wiszniewska, Joanna Peradzyńska

**Affiliations:** 1 Department of Paediatrics, Medical University of Warsaw, 02-091 Warsaw, Poland; agata.strozyk@wum.edu.pl (A.S.); daria.wiszniewska@gmail.com (D.W.); 2 Department of Pediatrics, Gastroenterology and Nutrition, Regional Specialized Children’s Hospital, University of Warmia and Mazury, 10-561 Olsztyn, Poland; elzbieta.jarocka@uwm.edu.pl; 3 Department of Epidemiology and Biostatistics, Medical University of Warsaw, 02-091 Warsaw, Poland; joanna.peradzynska@wum.edu.pl

**Keywords:** food allergy, children, diet diversity, feeding difficulties

## Abstract

**Background/Objectives:** This study aimed to evaluate diet diversity and feeding practices in toddlers with food allergy (FA) compared to healthy peers. **Methods:** This cross-sectional survey was conducted in Polish nurseries and included children aged 13–36 months with and without FA. Parents completed a questionnaire assessing feeding practices, anthropometric measurements, diet diversity using the Food Frequency Questionnaire, and feeding difficulties using the Montreal Children’s Hospital Feeding Scale. **Results:** Data from 388 children (predominantly from Warsaw and from families with high socioeconomic status) were analyzed. Among them, 61 (16%) had FA confirmed by a physician (however, an oral food challenge was performed only in one-third of cases). The proportion of underweight children (≤third percentile) was similar between the FA and non-FA groups (3.4 vs. 0.9%, respectively). Compared with the non-FA group, children with FA had significantly lower median overall diet diversity, food group diversity (≥nine food groups), food item diversity, and mean food allergen diversity. We found no difference in the proportion of children with feeding difficulties between the FA and non-FA groups (median = 18% vs. 13.5%). Although a lower proportion of children with FA had introduced cow’s milk, hen’s egg, tree nuts, nuts, and sesame compared with the non-FA group, only a minority had confirmed allergies to nuts, peanuts, and sesame. Most children with cow’s milk and hen’s egg allergy reintroduced baked milk (48.9%) and egg (40%). **Conclusions:** Children aged 13–36 months with FA are at risk of reduced overall diet diversity and over-restriction of potentially allergenic foods.

## 1. Introduction

Diet diversity (or variety) is defined as the number of different foods or food groups consumed over a given reference period [1]. Diversity within a specific food group—such as diversity of fruits and vegetables or diversity of food allergens––does not directly reflect overall diet diversity, but may be considered in combination with other measures to provide a more comprehensive assessment [1,2,3].

Diet diversity does not directly reflect diet quality; however, studies have demonstrated that greater diet variety is associated with higher nutrient intake, improved growth outcomes, and increased microbiome diversity [1,4]. A European Academy of Allergology and Clinical Immunology (EAACI) systematic review suggested that higher diet diversity in infancy may be linked to a reduced risk of allergic diseases later in life [1]. Furthermore, recent EAACI guidance on healthy complementary feeding practices for allergy prevention in developed countries emphasized that complementary foods introduced in infancy should be diverse, as greater diet diversity has been shown to lower the prevalence of food allergy by the age of 6–10 years [5].

In children with confirmed food allergy (FA), elimination of the relevant allergens from the child’s diet or from the breastfeeding mother’s diet constitutes first-line treatment [6,7,8]. While it is effective in preventing allergic reactions, it may be associated with delayed introduction of complementary foods, an increased risk of nutritional deficiencies, growth faltering, feeding difficulties, and social isolation [9,10]. Interestingly, many children with cow’s milk protein allergy (CMPA) and hen’s egg protein allergy (HEA) are able to tolerate baked milk and baked egg products [6,7]. Gradual reintroduction of allergenic foods, starting with baked forms and progressing according to food ladders, is a well-established approach to assessing and promoting tolerance acquisition in children with CMPA or HEA [6]. However, this strategy is often delayed or not implemented in clinical practice [8].

Although elimination diets introduced for suspected or confirmed FA in children may influence overall diet diversity, the available evidence remains limited. In one case-control study involving children aged 3–18 years with physician-diagnosed IgE-mediated FA, diet diversity was comparable to that observed in children with respiratory allergies consuming a regular diet (*n* = 160) [11]. In another cross-sectional study of children aged 8 to 27 months, diet variety was lower in those following a cow’s milk-free diet compared with those on an unrestricted diet (*n* = 126) [12].

Diet diversity during early childhood is influenced by a complex interplay of factors, including infant feeding practices (e.g., breastfeeding vs. formula feeding), timing of exposure to different foods and flavors, and socioeconomic status [13,14,15]. Early feeding practices may affect diet diversity; however, most research to date has focused on infants, while data on diet diversity among nursery-aged children remain limited. In Poland, the PITNUTS study assessed feeding practices in the general population of children aged 5 to 36 months [16]; however, it did not specifically address feeding practices in children with food allergies.

Current evidence on diet diversity and feeding practices in children with food allergy is limited and inconsistent. To address this gap, we conducted a cross-sectional survey aimed to evaluate overall diet diversity and feeding practices in children aged 13–36 months attending nurseries following elimination diets due to food allergy compared to healthy peers.

## 2. Materials and Methods

### 2.1. Study Design

This cross-sectional survey was conducted in a convenience sample of participants using both online and paper-based questionnaires.

### 2.2. Participants

Children aged 13–36 months attending public or private nurseries in Warsaw and Olsztyn were eligible for inclusion in the study, without restrictions regarding age (within the range), sex, ethnicity, nationality, parental education, or socioeconomic status.

The exposure group (FA group) consisted of children with physician-confirmed FA who were currently following an elimination diet, regardless of the diagnostic method used. In Polish nurseries, a medical certificate confirming FA is required to introduce an elimination diet. Therefore, all children had such documentation. For the purpose of this study we included children on elimination diets with OFC-confirmed FA, as well as those without OFC. For all children in the FA group, parents reported the diagnostic method, symptoms, and history of anaphylaxis. IgE- and non-IgE-mediated FA were not differentiated.

The control group (non-FA group) included healthy children on unrestricted diets recruited from the same nurseries as those in the exposure group.

Exclusion criteria were comorbidities significantly affecting dietary intake or nutritional status (e.g., celiac disease, inflammatory bowel disease), elimination diets for non-medical reasons (e.g., religious, ethical, vegetarian), enteral or parenteral nutrition, preterm birth (<37 weeks), or birth weight <2500 g.

No incentives for participation were provided, and respondents were informed that they could withdraw from the survey at any time.

### 2.3. Questionnaire Design

The questionnaire was developed by members of Food Allergy Section of the Polish Society of Gastroenterology, Hepatology, and Nutrition, including two pediatric gastroenterologists (A.H., E.J.-C.) and one dietitian (A.S.), all experienced in child nutrition and allergy research.

The questionnaire collected general demographic and anthropometric data (weight, height) as well as information on breastfeeding, formula feeding, consumption of plant-based beverages, introduction of complementary foods and potentially allergenic foods, and supplement use. In addition, two validated instruments were incorporated: the Food Frequency Questionnaire (FFQ-6) [17] and the Montreal Children’s Hospital Feeding Scale (MCH-FS) [18].

Anthropometric measures, including body weight and length/height, were reported by parents. Although direct measurement by researchers would have been preferable, prior to initiating the survey we confirmed with the nurseries that children’ weight and length/height are routinely measured by nursing staff and communicated to parents. Based on parent-reported values, a pediatrician (A.H.) calculated weight-for-age and length/height-for age percentiles using the World Health Organization (WHO) growth charts [19].

The frequency of consumption of 55 individual food items over the past month, grouped into 11 food categories, was assessed using the validated Food Frequency Questionnaire (FFQ-6) [17]. The FFQ-6 is a standardized instrument designed to evaluate habitual frequency of food consumption. For each food item, parents reported how often it was consumed using the following categories: never or almost never, less than once per week, once per week, 2–6 times per week, once per day, and several times per day. A 2017 systematic review confirmed that the FFQ is valid as a tool for estimating dietary intake in children aged 12–36 months [17].

Each parent was asked to complete the Polish version of the Montreal Children’s Hospital Feeding Scale (MCH-FS), a questionnaire validated for children aged 6 months to 6 years [18]. The MCH-FS consists of 14 items addressing various aspects of feeding, including parental concerns about feeding and child growth, parental feeding strategies, children’s oral motor and sensory functions, appetite, mealtime duration, and the impact of feeding on family relationships. Each item is rated on a seven-point Likert scale, and the total score is calculated by summing the individual item scores. The scoring range is 14 to 98 points, with a total score ≥46 indicating significant feeding difficulties.

Recruitment was conducted between March 2025 and June 2025 in nurseries located in Warsaw (A.H. and A.S.) and Olsztyn (E.J.-C.). For parents of children attending nurseries in Warsaw, the questionnaire was available online via the Warsaw Nurseries website and app, whereas in Olsztyn the majority of questionnaires were collected in paper form.

### 2.4. Outcomes

All study outcomes referred to feeding practices, overall diet diversity, and feeding difficulties in children aged 13–36 months and included the following:

▪ **nutritional status**, assessed using the WHO weight-for-age and length/height-for-age percentiles;
▪ **diet diversity measures**, including WHO Minimum Dietary Diversity [20], overall diet diversity, food group diversity, and food item diversity (Table 1);
▪ **specific food group diversity measures**, including food allergen diversity and fruit and vegetable diversity (Table 1);
▪ **percentage of children with feeding difficulties**, defined as those achieving a total score of at least 46 points on the MCH-FS [18];
▪ **feeding practices**, including: breastfeeding and formula feeding (i.e., duration and volume of exclusive and any breastfeeding, formula feeding, number of daytime and nighttime feeds), consumption of plant-based beverages, and complementary feeding practices (i.e., timing of solid foods introduction and introduction of potentially allergenic foods);
▪ **supplement use**.

**Table 1:** Diet and specific food group diversity measures.

Diet and Specific Food Group Diversity Measures	Definition
*Diet diversity measures*	
World Health Organization (WHO) Minimum Dietary Diversity [20]	▪ Defined for children aged 6–23 months. ▪ A sum of the number of food groups consumed from the following eight categories: breast milk; grains, roots, and tubers; legumes and nuts; dairy products; flesh foods (e.g., meat, fish, poultry, liver); eggs; vitamin A-rich fruits and vegetables; and other fruits and vegetables. ▪ Minimum dietary diversity is achieved when foods from at least five out of the eight groups are consumed.
Overall diet diversity (modified from Roduit et al. [21] and Venter et al. [2])	▪ A score out of 15 groups, based on consumption of the following groups: milk and natural milk beverages, other milk products, eggs, nuts, vegetables, fruits, potatoes, flakes, bread, pasta/rice, meat, fish, pulses, butter, and oils.
Food group diversity [1]	▪ A score out of 11 food groups, based on the consumption of the following groups: grains, dairy, eggs, meat, fish, vegetables, pulses, nuts and seeds, fruits, and fats. ▪ A diet is considered diverse when a child consumes foods from at least nine groups.
Food diversity [1]	▪ A score out of 43 foods assessed with FFQ (with the exclusion of sweets, salted snacks, and beverages).
*Specific food group diversity measures*	
Food allergen diversity [1,2]	▪ A score out of eight potentially allergenic foods, based on the introduction of the following: milk, eggs, wheat, fish, soy, peanuts, tree nuts, and sesame. ▪ A high food allergen diversity score was defined as the introduction of five or more potentially allergenic foods.
Fruit and vegetable diversity (modified from Venter et al. [2])	▪ A score out of five groups, based on consumption of the following groups: non-citrus fruits; citrus fruits (including kiwi and other citrus fruits); stone fruits and berry fruits; vegetables, avocado, and olives. ▪ A high fruit and vegetable diversity score was defined as the introduction of four or more groups.

FFQ, Food Frequency Questionnaire.

In the group of children with suspected or confirmed FA, we additionally collected the following data: family risk factors for reported FA symptoms, age and method of diagnosis, the specialist responsible for FA diagnosis and follow-up, the proportion of children with anaphylaxis, asthma, or atopic dermatitis, the proportion who had consulted with a dietitian, and the use of hypoallergenic formulas. Furthermore, we assessed the proportion of children who had reintroduced processed forms of milk and egg, as well as the proportion who achieved tolerance at each step of the milk and egg ladder. These data were reported separately for children with and without OFC-confirmed FA.

### 2.5. Ethics

This study was conducted in accordance with the Declaration of Helsinki. Ethical approval was obtained from the Ethical Committee Medical University of Warsaw (AKBE/281/2024). All participants provided voluntary informed consent to participate in the study and were informed that the data would be analyzed anonymously. Demographic data were collected only in broad categories.

### 2.6. Sample Size

There are a lack of epidemiological data in Poland on which to base a precise sample size calculation for this type of study. However, in a similar observational study conducted in children aged 3–18 years, which assessed diet diversity and body weight, 100 children with FA (exposure group) and 60 children with respiratory allergies (control group) were included [11].

In a comparable Polish study conducted in the under-3 age group in the Kuyavian-Pomeranian region, the FA group included 35 children, while the healthy control group consisted of 20 children [22].

Based on these data, a sample size of at least 200 children was considered sufficient for the purposes of the present study.

### 2.7. Statistical Analysis

Statistical analyses were performed using R software in version 4.4.2. Descriptive statistics were used to present the participants’ baseline characteristics. Nominal variables are presented as the number of patients (*n*) and percentages (%). Continuous variables are reported as means and standard deviation (SD) or medians with interquartile range (IQR), as appropriate (depending on data distribution). For all outcomes, mean or median differences (MDs) between groups, or relative risks (RRs), were calculated, each with corresponding 95% confidence intervals (CIs).

Comparisons and associations were assessed using appropriate tests depending on distribution normality and variance homogeneity. Normality was evaluated by Shapiro–Wilk test, along with skewness and kurtosis. All tests were two-tailed, with a significance level α = 0.05. Homogeneity of variances was assessed using Levene’s test. For comparisons between two groups, Student t test was applied for normally distributed data with equal variances, Welch t test was used for unequal variances, and Mann–Whitney U test was used for non-normally distributed variables. For categorical data, associations were tested using Pearson’s chi-square test or Fisher’s exact test, depending on expected frequencies. In analyses involving more than two groups, one-way ANOVA followed by Tukey’s post hoc test was used for parametric data, and Kruskal–Wallis test followed by Dunn’s post hoc test with Bonferroni correction was used for non-parametric data. Statistical significance was set at *p* < 0.05.

## 3. Results

Data from the online and paper-based questionnaires were available for 403 responders. Fifteen responses were excluded due to prematurity, very low birth weight, and/or substantial missing data. Therefore, data from 388 parents—61 with children on elimination diets due to FA and 327 with healthy children consuming unrestricted diet (non-FA)––were included in the final analysis.

### 3.1. Characteristics of Survey Population

The demographic characteristics are presented in Table 2. The majority of children were recruited in Warsaw (82.9%). Children in the FA group were slightly younger than those in the control group (median = 23 [IQR 19–31] months vs. 25 [IQR 20–31]) months; mean difference [MD] = −2.0 months, 95% CI: −4.0 to 0.0, *p* = 0.046). During their time at nurseries, most children (96.4%) consumed only meals prepared at the nursery. A higher proportion of children on an elimination diet due to FA consumed meals prepared at home and provided by parents, either partially (4.9%, *n* = 3 vs. 0.9%, *n* = 3) or fully (6.6%, *n* = 4 vs. 1.2%, *n* = 4), compared with the non-FA group. The FA and non-FA groups did not differ in other characteristics, including sex distribution, mean body weight and height, or parental economic status.

### 3.2. Characteristics of the Subgroup with Food Allergies

All children on an elimination diet had a physician-confirmed FA diagnosis (*n* = 61); however, only 39.3% had OFC-confirmed FA (Table S1). Almost half of parents reported using positive sIgE results to food allergens (45.9%) and/or clinical symptoms to confirm the FA diagnosis.

Most parents reported that their children had CMPA (77%), and almost half of children had hen’s egg protein allergy (49.2%). Allergies to other foods were less common, including peanuts (16.4%), soy (13.1%), nuts (11.5%), wheat (6.6%), and fish (3.3%).

**Figure 1:**
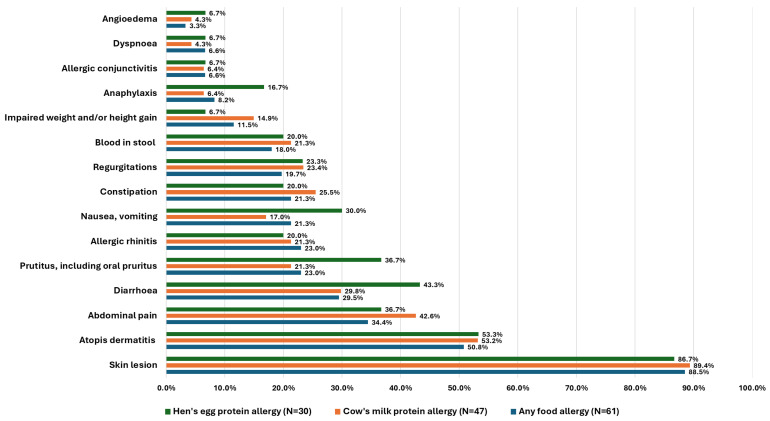
Symptoms of any food allergy, hen’s egg protein allergy, and cow’s milk protein allergy declared by parents.

Symptoms of food allergies declared by parents are presented in Figure 1.

#### 3.2.1. Children with Cow’s Milk Protein Allergy (CMPA)

A total of 47 respondents (77% of children with food allergy) reported that their children had a physician-diagnosed CMPA. According to FA diagnostic methods, only one third of parents of CMPA-children declared that CMPA diagnosis was confirmed by OFC (34%), nearly one third (29.8%) reported positive sIgE results for cow’s milk, and in almost half diagnosis was based mainly on clinical symptoms (44.7%). Only a small proportion of parents reported the use of component-resolved diagnostics or skin prick tests (Table S2).

CMPA was diagnosed by an allergologist in half of the children, with similar proportions in the OFC-confirmed and non-OFC subgroups (56.2 and 50.0%, respectively) (Table S3). More than one-third of children consulted a pediatrician (37.5% vs. 42.9%), while only a minority were diagnosed by a gastroenterologist (6.2% vs. 3.6%) or other specialist (3.6%). The median age of CMPA diagnosis was comparable between the two groups (5 vs. 6 months).

Most parents reported that their children had atopic dermatitis (75% in the OFC-confirmed CMPA subgroup vs. 54.8% in the non-OFC subgroup). Anaphylaxis (6.2 vs. 6.5%; RR = 1.0, 95% CI, 0.1 to 9.9, *p* > 0.999) and asthma (6.2 vs. 9.7%; RR = 0.7, 95% CI, 0.1 to 5.7, *p* > 0.999) were reported in only a few cases in both groups. Most children did not consult a dietitian in either the OFC-confirmed or non-OFC-confirmed CMPA groups (75% and 61.3%, respectively).

**Figure 2:**
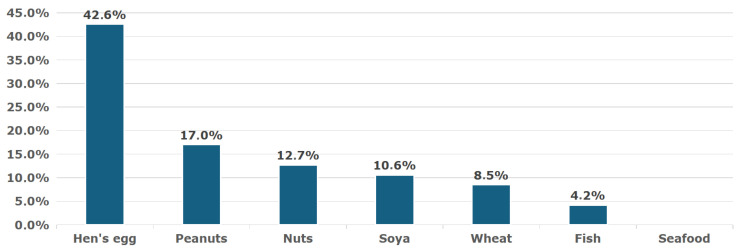
Concomitant food allergies in children with cow’s milk protein food allergy.

**Figure 3:**
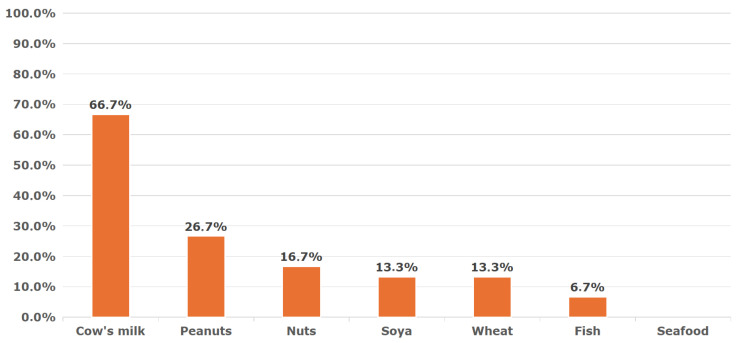
Concomitant food allergies in children with hen’s egg protein food allergy.

A significant proportion of children had coexisting food allergies to more than one food allergen—Figure 2 and Figure 3.

#### 3.2.2. Children with Hen’s Egg Protein Allergy (HEA)

A total of 30 parents (49% of children with food allergy) reported that their children had a physician-confirmed HEA. Among the reported diagnostic methods, positive sIgE results to hen’s egg were the most common (60.0%) (Table S4). Only one-third of children had OFC-confirmed HEA (33.3%) while a smaller proportion were diagnosed based on clinical symptoms (20%), component-resolved diagnostics (16.7%), or skin prick tests (10%).

In two thirds of children, HEA was confirmed by an allergologist, with similar proportions in the OFC-confirmed and non-OFC subgroups (60% vs. 70%, respectively). Less frequently, it was diagnosed by a pediatrician (40% vs. 20%, respectively) or other specialist (10%, only in the non-OFC group) (*p* = 0.449) (Table S5). The median age at HEA diagnosis was similar between the two subgroups (7.5 vs. 7 months; MD = 0.5, 95% CI: −4.0 to 3.0; *p* = 0.791).

Half of parents reported that their children had atopic dermatitis (50% in both subgroups). Anaphylaxis and asthma were reported only in a few cases (16.7% and 10%, respectively). About half of children did not consulted a dietitian in the OFC-confirmed and non-OFC-confirmed HEA groups (50% and 60%, respectively).

### 3.3. Nutritional Status (Weight and Length/Height-for-Age Percentiles)

No significant differences were observed between the FA and non-FA groups in the proportion of children at each WHO percentile cut-off points for weight-for-age and length/height-for-age (Table 3). Underweight (≤third percentile, WHO growth standards) was identified in a minority of children in both groups (3.4% in the FA group and 0.9% in the non-FA group). Similarly, stunting (≤third percentile) was infrequent (1.7% in the FA group and 3.5% in the non-FA group). In contrast, high body mass (>85th percentile) affected 25.4% of children in the FA group compared with 16.8% in the non-FA group; however, this difference was not statistically significant.

### 3.4. Diet Diversity

#### 3.4.1. WHO Minimum Dietary Diversity

The median WHO minimum dietary diversity score was statistically significantly lower in the FA group compared with the non-FA group (MD = 0.0 food groups; 95% CI: −1.0 to 0.0, *p* < 0.001), however, this difference was not clinically meaningful (Table 4). WHO minimum dietary diversity defined as ≥ five food groups consumed by a child at the time of investigation was achieved in nearly all children (98.4% vs. 100%), with no difference between groups.

#### 3.4.2. Overall Diet Diversity

Children on an elimination diet due to FA had a lower median overall diet diversity score compared with children on an unrestricted diet (MD = −1.00 food groups; 95% CI, −2.00; −1.00, *p* < 0.001).

#### 3.4.3. Food Group Diversity

A lower median food group diversity score was also observed in the FA group compared with the non-FA group (MD = −1.0 food groups, 95% CI: −1.0 to −1.0, *p* < 0.001). However, the extremely narrow confidence interval suggests that this finding should be interpreted with caution and may be clinically insignificant. The proportion of children achieving a diverse diet (≥nine food groups) was 15% lower in the FA group compared with the non-FA group (83.6% vs. 98.5%; RR = 0.9, 95% CI: 0.8 to 1.0, *p* < 0.001).

#### 3.4.4. Food Diversity

Children in the FA group had a significantly lower median food diversity score compared with those in the non-FA group (MD = −4.0 foods, 95% CI: −5.0 to −2.0, *p* < 0.001).

### 3.5. Specific Food Group Diversity

#### 3.5.1. Food Allergen Diversity

Children in the FA group had lower mean food allergen diversity scores compared with those in the non-FA group (MD = −0.9, 95% CI: −1.4 to −0.5, *p* < 0.001). The proportion of children with a high food allergen diversity score, defined as ≥ five potentially allergenic foods introduced, was 15% lower in the FA group compared to the non-FA group (72.1% vs. 85.0%; RR = 0.9, 95% CI: 0.7 to 1.0, *p* = 0.023).

#### 3.5.2. Fruit and Vegetable Diversity

No significant differences were observed in median fruit and vegetable diversity scores between the FA group and the non-FA group (MD = 0.0, 95% CI: 0.0 to 0.0, *p* = 0.622). High fruit and vegetable diversity, defined as ≥ four food groups, was achieved in both groups (95.1 vs. 93.6%).

### 3.6. Feeding Difficulties

No significant differences were observed between the FA group and the non-FA group in the proportion of children with feeding difficulties (*p* = 0.470), defined as a total score of MCH-FS ≥46. Nevertheless, the proportion of children with feeding difficulties was significant in both groups (median = 18 vs. 13.5%, *n* = 386; Table 4). No difference was observed between groups in the median MCH-FS total score (30.0 vs. 28.0, respectively, *p* = 0.117).

### 3.7. Breastfeeding, Formula Feeding, and Complementary Feeding Practices

#### 3.7.1. Any and Exclusive Breastfeeding

No significant differences were observed between the FA group and the non-FA group in the duration of any or exclusive breastfeeding (Table 5). Approximately one fifth of children were still breastfed in both groups (19.7%, *n* = 12 in the FA group; 21.1%, *n* = 61 in the non-FA group). The median number of daytime human milk feeds was similar in both groups (median = 5 vs. 4 feeds; MD = 1.0; 95% CI: 0.0 to −4.0, *p* = 0.056; *n* = 72). However, the mean number of nighttime feeds was higher in the FA group compared with the non-FA group (3.6 vs. 2.4 feeds; MD = −1.2; 95% CI: 0.2 to 2.1, *p* = 0.015; *n* = 70).

#### 3.7.2. Formula Feeding

There was no significant difference in the proportion of children who were formula-fed between the FA group and the non-FA group (37.7 vs. 30.3%, respectively, *n* = 389). However, the groups differed significantly regarding the type of human milk substitutes used.

Among formula-fed children, hypoallergenic human milk substitutes (amino acid formula [AAF], extensively hydrolyzed formula [EHF], and hypoallergenic formula [HA]) were used more frequently in the FA group compared with the non-FA group (13% [*n* = 3] vs. 0.0%, 47.8% vs. 2.1% [*n* = 2], and 4.3% [*n* = 1] vs. 0.0% respectively; *n* = 122). Conversely, young child formula was used less frequently in the FA group than in the non-FA group (21.7% [*n* = 5] vs. 92.7%; *n* = 122). Follow-on formula and goat’s milk formula were used more commonly in the FA group (8.7 [*n* = 2] vs. 2.1% [*n* = 2] and 4.3 [*n* = 1] vs. 3.1% [*n* = 3], respectively; *n* = 122).

The median daily intake of infant or hypoallergenic formula was similar in both groups (400 mL; *n* = 120), as was the median daily number of feeds (2 vs. 2.1; *n* = 120).

##### Hypoallergenic Formula Use in Children with Cow’s Milk Protein Allergy

One third of children with CMPA used human milk substitute (37.5 vs. 25.8%, in the OFC-confirmed and non-OFC groups, respectively; RR = 1.5, 95% CI: 0.6 to 3.5, *p* = 0.621) (Table S3). The median daily intake of human milk substitutes among children with CMPA was high in both groups (350 vs. 325 mL; MD = 25.0, 95% CI: −50.0 to 300.0; *p* = 0.414).

#### 3.7.3. Plant-Based Beverages

The proportion of children consuming plant-based beverages was nearly four times higher in the FA group compared with the non-FA group (47.5% vs. 13.1%, RR = 3.6, 95% CI: 2.5 to 5.3, *p* < 0.001; *n* = 388). Soy (9.8%, *n* = 6 vs. 2.4%, *n* = 8, RR = 4.0, 95% CI: 1.5 to 11.2, *p* = 0.013), almond (14.8%, *n* = 9 vs. 4.0%, RR = 3.7, 95% CI: 1.7 to 8.3, *p* = 0.003), and oat drinks (39.3% vs. 10.4%, RR = 3.8, 95% CI: 2.4 to 5.9, *p* < 0.001) were consumed significantly more frequently by children with FA compared with those without FA. Coconut drinks were reported only in the FA group (16.4%). Oat-based beverages were the most frequently consumed plant-based beverage in both groups (39.3 vs. 10.4%,; *n* = 388).

Among children who consumed plant-based beverages, fewer than half received calcium-fortified products (41.4% [12/29] vs. 46.5% [20/43]; *n* = 72). Children with FA received calcium-fortified beverages three times more frequently compared to those without FA (19.7% vs. 6.1%, RR = 3.2, 95% CI: 1.7 to 6.2, *p* = 0.001; *n* = 388). The median daily intake of plant-based beverages was similar in both groups (110 vs. 100 mL; *n* = 68). Among children with CMPA, half consumed plant-based beverages (55.3%, *n* = 47).

#### 3.7.4. Complementary Feeding Practices

The mean number of meals consumed per day was similar between the FA group and the non-FA group (4.4 vs. 4.6; MD = −0.2, 95% CI: −0.5 to 0.1; *n* = 389). No significant difference was observed between groups in the age of complementary feeding introduction (*p* = 0.066; *n* = 387). The majority of children in both groups were introduced to complementary foods between 17 and 26 weeks (62.3 vs. 60.1%). However, in approximately one third of children, the introduction of solid foods was delayed beyond 26 weeks (32.8 vs. 31.6%; *n* = 387).

##### Introduction of Potentially Allergenic Foods

The timing of introduction of potentially allergenic foods was similar in the FA and non-FA groups, with most children introduced to these foods concurrently with other solid foods (67.2 vs. 66.1%; *n* = 388). However, in approximately one third of children with FA, the introduction of potentially allergenic foods was delayed (27.9 vs. 33.0% *n* = 388) (Table 5).

With regard to specific allergens, the proportion of children who had cow’s milk introduced into their diet was 24% lower in the FA group compared to the non-FA group (73.8% vs. 96.6%, RR = 0.8, 95%CI, 0.7 to 0.9, *p* < 0.001). Similarly, eggs were introduced in 17% fewer children in the FA group compared with the non-FA group (82.0% vs. 98.8%; RR = 0.8, 95% CI: 0.7 to 0.9, *p* < 0.001). Peanuts and tree nuts were also introduced less frequently in the FA group compared with the non-FA group (50.8% vs. 70.0%, RR = 0.7, 95% CI: 0.6 to 0.9, *p* = 0.005; and 54.1% vs. 69.7%, RR = 0.8, 95%CI: 0.6 to 1.0, *p* = 0.025, respectively). Likewise, sesame was introduced 23% less often in the FA group compared with the non-FA group (52.5% vs. 68.5%, RR = 0.8, 95%CI: 0.6 to 1.0, *p* = 0.023).

### 3.8. Supplement Use

No significant differences were observed between the FA and non-FA groups in the use of dietary supplements, including vitamin D, docosahexaenoic acid (DHA), probiotics, calcium, iron, multivitamin and mineral preparations, and others (Table S6).

### 3.9. Association Between Selected Feeding Practices and Diet Diversity in Children with Food Allergy

In children with FA, a longer duration of any breastfeeding was associated with a higher median food allergen diversity score (*p* = 0.014) (Table S7). Children breastfed for 1.5–5 months (median = 6.0), 6–12 months (median = 7.0), and more than 12 months (median = 6.5) demonstrated greater food allergen diversity compared with those breastfed for less than 1 month (median = 4.0) or approximately 6 months (median = 4.5). All additional analyses evaluating associations between selected feeding practices and diet diversity, as well as feeding difficulties, are presented in Tables S7–S12.

### 3.10. Reintroduction Using Food Ladder

#### 3.10.1. Milk Ladder

The median age of baked milk reintroduction (12 months) and median time from CMPA diagnosis to milk reintroduction (6 months) were similar in children with OFC-confirmed CMPA and those without OFC (Table S3). For the majority of children, baked milk reintroduction was performed at home, with similar proportions in the two groups (92.3 vs. 100%; *n* = 47). Most children (81%; 38/47) started milk reintroduction using the milk ladder, and half of them achieved tolerance to baked and fried forms (cookies, muffins, and pancakes; 51.1%, 48.9%, and 55.3%, respectively). Tolerance to hard cheese was achieved by one third of children (29.8%), and 21.3% consumed fried/baked hard cheese without symptoms. A smaller proportion of children achieved tolerance to pasteurized milk or infant formula (23.4%) and/or raw fresh milk (10.6%).

#### 3.10.2. Egg Ladder

More than half of children (63.3% [*n* = 19/30]) started hen’s egg reintroduction using the egg ladder (Table S5). Although the difference between the OFC-confirmed and non-OFC HEA groups was not statistically significant, a slightly lower proportion of children in the OFC-confirmed group achieved tolerance to baked and fried forms of hen’s egg (cookies, muffins, and pancakes; 43.3%, 40.0%, and 40.0%, respectively). No children in the OFC-confirmed HEA group achieved tolerance to any cooked form of egg.

## 4. Discussion

### 4.1. Summary of the Main Results

This cross-sectional survey study conducted in Poland aimed to assess diet diversity and feeding practices in children aged 13–36 months on elimination diets due to FA who attend nurseries. A total of 388 participants were included, of whom 61 had FA. Although the diagnosis of FA was confirmed by a physician in all cases, only one third of children with FA had undergone an OFC.

A summary of the main findings is presented in Table 6. Briefly, no differences between the FA and non-FA groups were observed in the proportion of children with underweight and stunting. However, approximately 20% had a weight-for-age exceeding the 85th percentile. Children with FA had lower overall diet diversity, food group diversity (defined as ≥ nine consumed food groups), food diversity, and mean food allergen diversity compared with the non-FA group, while no differences were identified in WHO minimum diet diversity or fruit and vegetable diversity. Although no differences in feeding difficulties were found between the FA and non-FA groups, their proportion was high in both groups (18% and 13.5%, respectively).

Additionally, our study identified major inappropriate feeding practices in children with FA. Approximately one fifth of all children were still partially breastfed, with a higher number of night feeds observed in children with FA. One third of all children were fed with human milk substitutes, with no significant difference in the proportion of formula-fed children found between the groups. Plant-based beverages were consumed nearly four times more frequently by children with FA (almost half of respondents) compared with those without FA. Only half of the children who consumed plant-based beverages received calcium-fortified versions—three times more often in children with FA.

One third of parents reported delayed introduction of solid foods (>26 weeks of infant’s age) and potentially allergenic foods in both groups. Importantly, a lower proportion of children in the FA group had introduced cow’s milk, hen’s egg, tree nuts, peanuts, and sesame. Although the lack of cow’s milk and hen’s egg introduction can, at least partially, be explained by the coexistence of CMPA and/or HEA and allergy to tree nuts, peanuts, and sesame, FA was reported only in a minority of children.

About half of children with CMPA and 40% of children with HEA declared achievement of tolerance to baked forms of the respective food allergens.

### 4.2. Comparison to Other Studies, Systematic Reviews, and Guidelines

#### 4.2.1. Diet Diversity in Children with FA

In children older than 12 months, the diet should be aligned with healthy family meals and include a variety of nutrient-dense, fresh, minimally processed, home-cooked, and predominantly plant-based foods [5,23]. A more diverse diet may be associated with higher intake of immunomodulatory, nutrient-rich foods, increased gut microbial diversity, and broader exposure to dietary antigens, which may contribute to the prevention of atopic and allergic diseases [1]. Although the lower diet diversity observed in our study may partly reflect necessary elimination of confirmed allergens, we also identified parental over-restriction of other allergens (e.g., tree nuts, peanuts, sesame) without confirmed FA.

Current guidelines from major pediatric and allergic societies recommend against delaying the introduction of food allergens. It is suggested that peanuts in age-appropriate forms and well-cooked egg should be introduced at the start of complementary feeding, any time from 4 months of age, as a strategy to reduce the risk of developing allergy later in life [24,25]. A 2023 umbrella systematic review summarizing evidence from 32 systematic reviews found moderate-quality evidence supporting the introduction of peanut and egg between 4 and 11 months of age to prevent FA [26]. A recent UK population-based birth cohort study reported that, although most potentially allergenic foods were introduced between 6 and 9 months of age, the introduction of egg and nuts was delayed in a substantial proportion of children beyond 12 months of age (35% and 16%, respectively; *n* = 139) [27]. Furthermore, infants with a family history of allergy were more likely to lack certain foods in their diet due to parental concerns about allergy.

Most parents reported atopic dermatitis and other skin manifestations as the primary symptoms of their child’s food allergy. In a South Korean study conducted in children with atopic dermatitis aged 1 month to 18 years, dietary restriction without medical evaluation, based solely on parental judgement, was introduced in 39.7% of children (*n* = 191) [28]. A recent systematic review of 10 randomized controlled trials (*n* = 599) showed that dietary restriction in children with mild to moderate atopic dermatitis resulted in only slight, clinically non-significant improvements in eczema severity, pruritus, and sleeplessness [29]. These findings emphasize the need of appropriate nutritional education and timely referral to a dietitian trained in food allergy management to ensure adequate elimination of relevant allergens while avoiding unnecessary dietary restrictions [9,10]. Nevertheless, our study also revealed that most parents had not consulted a dietitian regarding their child’s FA, which may reflect both limited awareness and limited access to dietetic services in Poland.

#### 4.2.2. Human Milk Substitutes After One Year of Life

A high intake of infant and hypoallergenic formula observed in the studied population may contribute to reduced diet diversity. In our study, one third of children continued formula feeding beyond 12 months of age. The European Society of Paediatric Gastroentereology, Hepatology, and Nutrition (ESPGHAN) does not recommend the routine use of young child formula in children aged 1–3 years but acknowledges its potential role in specific populations as a part of a strategy to increase intake of iron, vitamin D, and polyunsaturated fatty acids [30]. Similarly, the 2025 National Institute for Health and Care Excellence (NICE) guidelines on maternal and child nutrition state that infant formula is not required beyond 1 year of age [23].

The continuation of hypoallergenic formula consumption in children with FA aged 13 months and older remains a matter of debate. According to recent EAACI guidance on complementary feeding, in children with FA or growth faltering, continuation of infant or hypoallergenic formula may be considered [5]. Similarly, British Society for Allergy and Clinical Immunology (BSACI) guidelines recommend that, in children under 2 years of age with CMPA who are not breastfed, a suitable substitute milk should be used, while in older children this is no longer necessary, whenever the child has adequate intake of energy, protein, calcium, and vitamins [31].

#### 4.2.3. Plant-Based Beverages After 1 Year of Life

In our study, for some children human milk substitutes were replaced with plant-based beverages. Plant-based beverages have been widely criticized as nutritionally inadequate for supporting growth in young children and are generally not recommended in children under 3 years of age with CMPA [32]. Nevertheless, parental interest in using plant-based beverages instead of commercial formulas is growing. Therefore, experts suggest that fortified plant-based beverages may be considered and successfully introduced in a carefully selected group of children over 1 year of age who meet specific nutritional criteria, i.e., a well-balanced and diverse diet, absence of feeding difficulties and micronutrient deficiencies, consumption of at least two thirds of their daily energy from solid foods, and intake of no more than 500 mL of milk substitute per day [33]. In clinical practice, it should be emphasized to parents that only calcium-fortified plant-based beverages are appropriate, as our study found that fewer than half of children received fortified products [33,34].

### 4.3. Strengths and Limitations

To our knowledge, this is the first study to assess diet diversity and feeding practices in children aged 13–36 months with physician-confirmed FA compared to healthy children recruited from the general population (nurseries). Therefore, these findings may be applicable not only to children with severe allergic symptoms typically referred to academic hospitals, but also to the broader population of children with mild and moderate allergic symptoms.

A significant proportion of parents reported that their children had not undergone an OFC to confirm the diagnosis of FA. Although OFC may be omitted in cases with very high specific IgE levels, large skin prick test wheals, or a clear history of anaphylaxis [6], we cannot exclude the possibility that some cases in our study were misdiagnosed. However, these findings reflect the real-world situation in Poland, where a large proportion of FA diagnoses are not confirmed by OFC. In a 2020 survey conducted by our research team, 72.6% of physicians reported not using OFC to confirm FA [35]. Nevertheless, elimination diets excluding common allergens are frequently introduced in children, most often due to strong parental beliefs regarding the causal relationship between food consumption and symptoms [36]. To minimize this potential bias, we analyzed and reported outcomes separately for children with OFC-confirmed and non-OFC confirmed FA; however, no significant differences were observed between these subgroups.

The relatively large study sample and the use of standardized, validated tools for identifying feeding difficulties (MCH-FS) and assessing dietary diversity (FFQ-6) are important strength of this study. However, we used convenience sampling, which likely attracted parents more interested in infant feeding or those perceiving feeding issues in their children. In addition, the survey included only children from two large Polish cities (Warsaw and Olsztyn) with parents reporting high socioeconomic status. Therefore, the findings may not be generalizable to children from smaller towns, rural areas, or families with lower socioeconomic status. Due to its cross-sectional nature, our study may also be subject to non-response bias, as participants who agreed to contribute may differ systematically from those who declined. Furthermore, parental self-reported data may be affected by recall bias—particularly for retrospective information such as breastfeeding duration or age of complementary food introduction––and by social desirability bias leading to overreporting of behaviors perceived as healthy.

Finally, as with any cross-sectional study, the nature of this study precludes any causal interferences. Nevertheless, we found strong correlations between physician-declared FA and several feeding practices, including higher consumption of plant-based beverages, delayed introduction of potentially allergenic foods, and lower diet diversity.

## 5. Conclusions

This study confirms that children aged 13–36 months on elimination diets due to FA are at risk of reduced diet diversity and food allergen diversity. The WHO minimum dietary diversity achieved by the majority of children with FA suggests that the nutritionally adequacy of their diet may not be impaired. Additionally, our findings clarified major feeding issues in children with FA, including delayed introduction of potentially allergenic foods, over-restriction of potentially allergenic foods, replacing human milk substitutes with plant-based beverages, and a high number of night breastfeeds. Individualized nutritional counseling for children with FA should be promoted to support appropriate allergen introduction, ensure adequate diet variety, and prevent over-restriction. Further research in populations with lower socioeconomic status, as well as more in-depth studies assessing potential factors influencing diet diversity, including parents’ dietary patterns, are needed.

## Figures and Tables

**Table 2:** Characteristics of the study population.

Variable	Total (*n* = 388)	With Food Allergy (*n* = 61)	Without Food Allergy (*n* = 327)	MD (95% CI)	*p*
Recruitment site: Warsaw *n* (%)	321 (82.7)	54 (88.5)	267 (81.7)	-	0.282
Age, months, median (IQR)	25.0 (19.0;31.0)	23.0 (16.0;30.0)	25.0 (20.0;31.0)	−2.0 (−4.0;0.0)	**0.046**
Gender: male, *n* (%)	186 (47.9)	36 (59.0)	150 (45.9)	-	0.081
Body weight, kg, mean ± SD *	12.7 ± 2.1	12.5 ± 2.3	12.7 ± 2.0	−0.2 (−0.7;0.4)	0.624
Height, cm, median (IQR) **	90.0 (84.0;94.0)	88.0 (83.0;94.5)	90.0 (85.0;94.0)	−2.0 (−4.0;1.0)	0.213
Meals in nurseries, *n* (%)					
Only foods provided by the nursery	374 (96.4)	54 (88.5)	320 (97.9)	-	**0.003**
Foods provided by the nursery and delivered by parents (prepared at home)	6 (1.5)	3 (4.9)	3 (0.9)		
Only foods delivered by parents (prepared at home)	8 (2.1)	4 (6.6)	4 (1.2)		
Economic situation, *n* (%)					
Sufficient for daily functioning, allowing for savings	278 (71.6)	43 (70.5)	235 (71.9)	-	0.402
Sufficient for daily functioning, but not for savings	84 (21.6)	14 (23.0)	70 (21.4)		
Sufficient for daily functioning, but requires giving up certain expenses	18 (4.6)	2 (3.3)	16 (4.9)		
Requires a very frugal lifestyle to save for larger expenses	4 (1.0)	0 (0.0)	4 (1.2)		
Money is only sufficient for basic needs	4 (1.0)	2 (3.3)	2 (0.6)		

SD—standard deviation, IQR—interquartile range, MD— mean or median difference (allergy vs. no allergy), CI—confidence interval. Groups were compared by Student *t* test (body weight), Mann–Whitney U test (age, height), Pearson’s chi-square test (sex), or Fisher’s exact test (economic situation). * Data available for *n* = 375 children in the total group (*n* = 59 in the allergy group and *n* = 316 in the no allergy group). ** Data available for *n* = 372 children in the total group (*n* = 59 in the allergy group and *n* = 313 in the no allergy group).

**Table 3:** Proportion of children at each WHO percentile cut-off points for weight-for-age and height-for-age.

Variable	Total (*n* = 388)	With Food Allergy (*n* = 61)	Without Food Allergy (*n* = 327)	*p*
*Weight-for-age,* *percentile, n (%)*				
≤3	5 (1.3)	2 (3.4)	3 (0.9)	0.178
3–15	21 (5.6)	2 (3.4)	19 (6.0)	0.551
15–85	281 (74.9)	40 (67.8)	241 (76.3)	0.225
85–97	49 (13.1)	12 (20.3)	37 (11.7)	0.111
>97	19 (5.1)	3 (5.1)	16 (5.1)	>0.999
*Height-for-age,* *percentile, n (%)*				
≤3	12 (3.2)	1 (1.7)	11 (3.5)	0.700
3–15	29 (7.8)	4 (6.8)	25 (8.0)	0.958
15–85	204 (54.8)	36 (61.0)	168 (53.7)	0.370
85–97	72 (19.4)	10 (16.9)	62 (19.8)	0.741
>97	55 (14.8)	8 (13.6)	47 (15.0)	0.929

Groups were compared by Pearson’s chi-square test or Fisher’s exact test, as appropriate.

**Table 4:** Diet diversity, specific food group diversity, and feeding difficulties in children with and without food allergy (*n* = 388).

Measure of Diet Diversity	With Food Allergy (*n* = 61)	Without Food Allergy (*n* = 327)	MD/RR (95% CI)	*p*
*Diet diversity measures*				
WHO minimum dietary diversity [0–8], median (IQR)	7.0 (6.0;7.0)	7.0 (7.0;7.0)	0.0 (−1.0;0.0)	**<0.001**
WHO minimum dietary diversity, diverse (≥5), *n* (%)	60 (98.4)	327 (100.0)	1.0 (1.0;1.0)	0.157
Overall diet diversity [0–15], median (IQR)	13.0 (11.0;14.0)	14.0 (14.0;15.0)	−1.0 (−2.0;−1.0)	**<0.001**
Food group diversity [0–11], median (IQR)	10.0 (9.0;11.0)	11.0 (10.0;11.0)	−1.0 (−1.0;−1.0)	**<0.001**
Food group diversity, diverse (≥9), *n* (%)	51 (83.6)	322 (98.5)	0.9 (0.8;1.0)	**<0.001**
Foods diversity (43 products) [0–43], median (IQR)	32.0 (28.0;36.0)	36.0 (32.0;39.0)	−4.0 (−5.0;−2.0)	**<0.001**
*Specific food groups diversity measures*				
Food allergen diversity [0–8], mean ± SD	5.6 ± 2.0	6.5 ± 1.6	−0.9 (−1.4;−0.5)	**<0.001**
Food allergen diversity, diverse (≥5), *n* (%)	44 (72.1)	278 (85.0)	0.9 (0.7;1.0)	**0.023**
Fruit and vegetable diversity [0–5], median (IQR)	5.00 (4.0;5.0)	5.00 (5.0;5.0)	0.0 (0.0;0.0)	0.622
Fruit and vegetable diversity, diverse (≥4), *n* (%)	58 (95.1)	306 (93.6)	1.0 (1.0;1.1)	>0.999
*Feeding difficulties*				
Children with feeding difficulties, *n* (%) *^†^	11 (18.0)	44 (13.5)	1.3 (0.7;2.4)	0.470
MCH–FS total score [14–98], median (IQR) *	30.0 (23.0;38.0)	28.0 (22.0;37.0)	2.0 (−1.0;5.0)	0.117

SD—standard deviation, IQR—interquartile range, MD—mean or median difference (allergy vs. no allergy), CI—confidence interval. Groups were compared by Student t test (Food Frequency Questionnaire (FFQ)—potentially allergenic food), Mann–Whitney U test (all other variables), Pearson’s chi-square test (MCH-FS, existing difficulties; FFQ—potentially allergenic food, diverse) or Fisher’s exact test (FFQ—minimal diversity WHO, diverse; FFQ—fruits and vegetables, diverse; FFQ—product group diversity, diverse). * Data available for *n* = 386 children (*n* = 61 in the allergy group and *n* = 325 in the no allergy group). ^†^ ≥46 total score on the MCH-FS.

**Table 5:** Feeding practices of children with and without food allergy (*n* = 388).

Variable	With Food Allergy (*n* = 61)	Respondents, *n*	Without Food Allergy (*n* = 327)	Respondents, *n*	MD/RR (95% CI)	*p*
*Breastfeeding duration*						
Duration of any breastfeeding, *n* (%)		59		289		
<1 month	10 (16.9)		29 (10.0)		-	0.427
1.5–5 months	10 (16.9)		57 (19.7)			
6 months	4 (6.8)		22 (7.6)			
6–12 months	19 (32.2)		78 (27.0)			
>12 months	16 (27.1)		103 (35.6)			
Duration of exclusive breastfeeding, *n* (%)		53		275		
<1 month	12 (22.6)		73 (26.5)		-	0.418
2–5 months	11 (20.8)		50 (18.2)			
6 months	21 (39.6)		125 (45.5)			
≥12 months	9 (17.0)		27 (9.8)			
Number of breastfeedings per day, median (IQR) *	5.0 (4.0;9.0)	11	4.0 (3.0;6.0)	61	1.0 (0.0;4.0)	0.056
Number of breastfeedings at night, mean ± SD	3.6 ± 1.6	12	2.4 ± 1.5	58	1.2 (0.2;2.1)	**0.015**
*Formula feeding*						
Fed with human milk substitute, *n* (%)	23 (37.7)	61	99 (30.3)	327	1.25 (0.9;1.8)	0.319
Type of human milk substitute, *n* (%) *		23		99		
AAF	3 (13.0)		0 (0.0)		-	**<0.001**
EHF	11 (47.8)		2 (2.1)			
HA	1 (4.3)		0 (0.0)			
Young child formula	5 (21.7)		89 (92.7)			
Follow-on formula	2 (8.7)		2 (2.1)			
Goat milk formula	1 (4.3)		3 (3.1)			
Human milk substitute, intake per day, mL, median (IQR)	400.0 (260.0;520.0)	23	400.0 (240.0;540.0)	97	0.0 (−80.0;120.0)	0.794
Number of feedings with human milk substitute per day, mean ± SD	2.0 ± 1.1	22	2.1 ± 1.1	98	−0.1 (−0.6;0.4)	0.697
*Plant-based beverages*						
Fed with plant drink, *n* (%)	29 (47.5)	61	43 (13.1)	327	3.6 (2.5;5.3)	**<0.001**
Type of plant drink, *n* (%)		61		327		
Soya	6 (9.8)		8 (2.4)		4.0 (1.5;11.2)	0.013
Almond	9 (14.8)		13 (4.0)		3.7 (1.7;8.3)	0.003
Oat	24 (39.3)		34 (10.4)		3.8 (2.4;5.9)	<0.001
Rice	0 (0.0)		1 (0.3)		-	>0.999
Coconut	10 (16.4)		0 (0.0)		-	**<0.001**
Fed with fortified plant drink, *n* (%)	12 (19.7)	61	20 (6.1)	327	3.2 (1.7;6.2)	0.001
Plant drink intake per day, mL, median (IQR)	110.0 (87.5;206.3)	28	100.0 (42.5;170.0)	40	10.0 (0.0;100.0)	0.197
*Complementary feeding practices*						
Number of meals per day, mean ± SD	4.4 ± 1.0	61	4.6 ± 1.1	327	−0.2 (−0.5;0.1)	0.144
Age of complementary foods introduction, *n* (%)		61		326		
<17 weeks	3 (4.9)		27 (8.3)		-	0.666
17–26 weeks	38 (62.3)		196 (60.1)			
>26 weeks	20 (32.8)		103 (31.6)			
Introduction of potentially allergenic foods (e.g., milk, egg, wheat, nuts), *n* (%)		61		327		
Aligned with other complementary foods	41 (67.2)		216 (66.1)		-	0.056
Delayed compared to other complementary foods	17 (27.9)		108 (33.0)			
Not introduced yet	3 (4.9)		3 (0.9)			
Introduction of food allergens at timepoint of investigation, *n* (%)		61		327		
Milk	45 (73.8)		316 (96.6)		0.8 (0.7;0.9)	**<0.001**
Eggs	50 (82.0)		323 (98.8)		0.8 (0.7;0.9)	**<0.001**
Wheat	57 (93.4)		319 (97.6)		0.96 (0.9;1.0)	0.103
Fish	58 (95.1)		318 (97.2)		0.98 (0.9;1.0)	0.412
Soya	34 (55.7)		172 (52.6)		1.1 (0.8;1.4)	0.756
Peanuts	31 (50.8)		229 (70.0)		0.7 (0.6;0.9)	**0.005**
Nuts	33 (54.1)		228 (69.7)		0.8 (0.6;1.0)	**0.025**
Sesame	32 (52.5)		224 (68.5)		0.8 (0.6;1.0)	**0.023**

SD—standard deviation, IQR—interquartile range, MD—mean or median difference (allergy vs. no allergy), RR—relative risk (allergy vs. no allergy), CI—confidence interval. Groups were compared by Student t test (number of breastfeedings at night, number of feedings with infant formula/breast milk substitute per day, number of meals per day), Mann–Whitney U test (number of breastfeedings per day, infant formula/breast milk substitute intake per day, plant drink intake per day), Pearson’s chi-square test (all other variables), or Fisher’s exact test (type of infant formula/breast milk substitute, rice, coconut, and other type of plant drinks and milk, eggs, gluten, fish introduced to diet). * Proportion of type of infant formula/breast milk substitute calculated based on all children fed with infant formula/breast milk substitute.

**Table 6:** Summary of the main findings.

Summary of Main Findings	
Children with Food Allergy (FA) Versus Without Food Allergy (Non-FA)	
**Underweight**	▪ No difference between group (weight-for-age < 3rd percentile: 3.4 vs. 0.9%).
**Diet diversity**	▪ **Lower median overall diet diversity (≥9 food groups), food groups diversity, foods diversity and mean food allergen diversity in the FA group.** ▪ No difference between groups WHO minimum dietary difference & fruit and vegetable diversity.
**Any feeding difficulties** **(MCH-FS ≥ 46 points)**	▪ No difference in any feeding difficulties between groups ▪ **High proportion of children with feeding difficulties in both groups (18 vs.13.5%)**
**Breastfeeding**	▪ No difference in duration of any and exclusive breastfeeding between groups. ▪ Higher mean number of night feeds in FA group (3.58 feeds)
**Human-milk substitutes**	▪ No difference in the proportion of formula-fed children; one third of children fed with human milk substitute (37.7 vs. 30.3). ▪ One third of children consumed young child formula in non-FA group (27.2%). ▪ High median daily intake of human milk substitute—400 mL. ▪ **One third of children with CMPA used EHF or AAF (29.7%).**
**Plant-based beverages**	▪ **Nearly 4-times higher consumption rate in FA group (47.5 vs. 13.1%).** ▪ Oat-based beverage was most frequently consumed in both group (82.8% vs. 79.1%). ▪ Nearly 3-times more frequently children with FA received a calcium-fortified beverage compared to non-FA group (19.7% vs 6.1%).
**Age of complementary foods introduction**	▪ No difference in the age of complementary foods introduction between groups; one third of children in both groups had delayed introduction of solid foods (>26 weeks: 32.8 vs. 31.6%).
**Delayed i** **ntroduction of potentially allergenic foods**	▪ One third of children in both groups had delayed introduction of potentially allergenic foods (27.9 vs. 33%). ▪ **Lower proportion of children in FA group had introduced cow’s milk, egg, peanuts, tree nuts and sesame.**
**Supplement use**	▪ No difference between groups.
**Milk/egg** **ladders**	▪ Among children with cow’s milk protein allergy 53.3% achieved tolerance to pancake and 48.9% to muffin. ▪ In children with hen’s egg protein allergy 40% achieved tolerance to fried forms and muffin, and 43.3% to cookie.

AAF, amino acid formula; CMPA, cow’s milk protein allergy; EHF, extensively hydrolyzed formula; FA, food allergy; non-FA, no food allergy; MCH-FS, Montreal Children’s Hospital Feeding Scale; WHO, World Health Organization.

## Data Availability

The original contributions presented in the study are included in the article/Supplementary Material, further inquiries can be directed to the corresponding author.

## Supplementary Materials

The following supporting information can be downloaded at: https://www.mdpi.com/article/10.3390/nu17203212/s1, Table S1. Characteristics of children with any food allergy (*n* = 61). Table S2. Characteristics of subgroup of children with cow’s milk proteins allergy (*n* = 47). Table S3. Association between diagnosis based on oral food challenge and selected feeding practices in subgroup of children with cow’s milk proteins allergy (*n* = 47). Table S4. Characteristics of subgroup of children with hen’s eggs proteins allergy (*n* = 30). Table S5. Association between diagnosis based on oral food challenge and selected feeding practices in subgroup of children with hen’s egg proteins allergy (*n* = 30). Table S6. Supplement use in children with and without food allergy (*n* = 388). Table S7. Association between any breastfeeding duration and diet and specific food group diversity in children with food allergy (*n* = 61). Table S8. Association between exclusive breastfeeding duration and diet and specific food group diversity in children with food allergy (*n* = 61). Table S9. Association between feeding difficulties, diet and specific food groups diversity and feeding with human milk substitutes in children with food allergy (*n* = 61). Table S10. Association between feeding difficulties, diet and specific food groups diversity and consuming plant-based beverages in children with food allergy (*n* = 61). Table S11. Association between declared food allergy symptoms and delay in complementary foods introduction in children with food allergy (*n* = 61). Table S12. Association between declared food allergy symptoms and delay in introduction of potentially allergenic foods in children with food allergy (*n* = 61).

## Abbreviations

The following abbreviations are used in this manuscript:

AAF	Amino acid formula
BSACI	British Society for Allergy and Clinical Immunology
CI	Confidence interval
CMPA	Cow’s milk protein allergy
DHA	Docosahexaenoic acid
EAACI	European Academy of Allergology and Clinical Immunology
ESPGHAN	European Society of Pediatric Gastroenterology, Hepatology, and Nutrition
EHF	Extensive hydrolyzed formula
FA	Food allergy
FFQ	Food Frequency Questionnaire
HF	Hypoallergenic formula
HEA	Hen’s egg protein allergy
IQR	Interquartile range
MD	Mean or median difference
MCH-FS	Montreal Children’s Hospital Feeding Scale
NICE	National Institute for Health and Care Excellence
Non-FA	No food allergy
OFC	Oral food challenge
RR	Relative risk
SD	Standard deviation
WHO	World Health Organization
