# Supplementary material for: Diet Diversity and Feeding Practices in Toddlers with and Without Food Allergy—A Cross-Sectional Study
Source: Nutrients. 2025 Oct 13;17(20):3212. doi: 10.3390/nu17203212 (PMC12566712; doi:10.3390/nu17203212)
Supplement: Supplementary file 1 [file nutrients-17-03212-s001.zip › nutrients-3914178-supplementary.pdf]

Supplementary File S1. Supplementary Tables S1–S12.

Table S1. Characteristics of children with any food allergy (n=61).

Variable	Children with any food allergy
Food allergy symptoms, n (%)	
Skin lesions	54 (88.5)
Atopic dermatitis	31 (50.8)
Abdominal pain Angioedema	21 (34.4)
Diarrhea	18 (29.5)
Pruritus, including oral pruritus	14 (23.0)
Allergic rhinitis	14 (23.0)
Nausea, vomiting	13 (21.3)
Constipation	13 (21.3)
Regurgitation/spitting up	12 (19.7)
Blood in stool	11 (18.0)
Impaired weight and/or height gain	7 (11.5)
Anaphylaxis	5 (8.2)
Dyspnoea	4 (6.6)
Allergic conjunctivitis	4 (6.6)
Angioedema	2 (3.3)
Declared Food Allergy, n (%)	
Milk	47 (77.0)
Eggs	30 (49.2)
Peanuts	10 (16.4)
Soya	8 (13.1)
Nuts	7 (11.5)
Gluten	4 (6.6)
Fish	2 (3.3)
Seafood	0 (0.0)
Method of Confirmation the Food allergy diagnosis, n (%)	
sIgE level	28 (45.9)
Diagnosis based solely on clinical symptoms	25 (41.0)
Oral Food Challenge	24 (39.3)
Component-resolved diagnostics	6 (9.8)
Skin tests	5 (8.2)

Healthcare specialist who diagnosed food allergy, n (%)	
Alergologist	38 (62.3)
Pediatrician	36 (59.0)
Gastroenterologist	3 (4.9)
General practitioner	3 (4.9)
Other	6 (9.8)
Family history of allergic diseases, n (%)	32 (52.5)
Allergic rhinitis	15 (24.6)
Food allergy	14 (23.0)
Atopic dermatitis	11 (18.0)
Asthma	7 (11.5)
Atopic dermatitis, n (%)	32 (52.5)
Anaphylaxis, n (%)	6 (9.8)
Asthma, n (%)	5 (8.2)
Dietary consultation, n (%)	
None	41 (67.2)
One time	9 (14.8)
2-3 times	5 (8.2)
3-4 times	3 (4.9)
Above 4 times	3 (4.9)
Children fed with human milk substitutes, n (%)	14 (23.0)
Daily intake of human milk substitutes, ml, mean ± SD	366.3±170.0

SD – standard deviation

Table S2. Characteristics of subgroup of children with cow's milk proteins allergy (n=47).

**Variable**	**n (%)**
Diagnostic methods	
Oral food challenge	16 (34.0)
sIgE level	14 (29.8)
Component-resolved diagnostics	4 (8.5)
Skin-prick test	2 (4.3)
Diagnosis based solely on clinical symptoms	21 (44.7)
Children fed with hydrolyzed or amino-acid formula	14 (29.8)
Milk reintroduction (milk ladder) at timepoint of investigation	
Baked (cookie)	24 (51.1)
Baked (muffin)	23 (48.9)
Fried (pancake)	26 (55.3)
Fried or baked yellow cheese	10 (21.3)
Hard yellow cheese	14 (29.8)
Natural yogurt	16 (34.0)
Pasteurized milk or infant formula	11 (23.4)
Raw fresh milk	5 (10.6)
Children consuming plant-based beverages	26 (55.3)
Food allergy symptoms	
Skin lesions	42 (89.4)
Pruritus, including oral pruritus	10 (21.3)
Angioedema	2 (4.3)
Allergic rhinitis	10 (21.3)
Allergic conjunctivitis	3 (6.4)
Abdominal pain	20 (42.6)
Nausea, vomiting	8 (17.0)
Diarrhea	14 (29.8)
Regurgitation/spitting up	11 (23.4)
Constipation	12 (25.5)
Blood in stool	10 (21.3)
Impaired weight and/or height gain	7 (14.9)
Atopic dermatitis	25 (53.2)
Anaphylaxis (systemic reaction)	3 (6.4)
Dyspnoea	2 (4.3)

Table S3. Association between diagnosis based on oral food challenge and selected feeding practices in subgroup of children with cow's milk proteins allergy (n=47).

	**Oral Food Challenge**			
Variable	Yes	No	MD/RR (95% CI)	p
Duration of exclusive breastfeeding, n (%)				
<1 month	5 (33.3)	4 (15.4)	-	0.088
2-5 months	5 (33.3)	5 (19.2)		
6 months	5 (33.3)	10 (38.5)		
≥12 months	0 (0.0)	7 (26.9)		
Age of complementary foods introduction, n (%)				
<17 weeks	1 (6.2)	1 (3.2)	-	0.877
17-26 weeks	10 (62.5)	22 (71.0)		
>26 weeks	5 (31.2)	8 (25.8)		
Introduction of potentially allergenic foods, n (%)				
Aligned with other complementary foods	9 (56.2)	22 (71.0)	-	0.275
Delayed compared to other complementary foods	7 (43.8)	7 (22.6)		
Not yet introduced	0 (0.0)	2 (6.5)		
Declared food allergy, n (%)				
Milk	16 (100.0)	31 (100.0)	-	>0.999
Eggs	8 (50.0)	12 (38.7)	1.29 (0.7;2.5)	0.667
Peanuts	4 (26.7)	4 (15.4)	1.73 (0.5;6:0)	0.434
Nuts	3 (20.0)	2 (7.7)	2.60 (0.5;13.9)	0.336
Wheat	2 (13.3)	2 (7.7)	1.7 (0.3;11.1)	0.615
Fish	2 (13.3)	0 (0.0)	-	0.128
Seafood	0 (0.0)	0 (0.0)	-	-
Soya	1 (6.7)	4 (14.8)	0.45 (0.1;3.7)	0.639
Healthcare specialist who diagnosed food allergy, n (%)				
Alergologist	9 (56.2)	14 (50.0)	-	0.925
Gastroenterologist	1 (6.2)	1 (3.6)		
Pediatrician	6 (37.5)	12 (42.9)		
Other	0 (0.0)	1 (3.6)		
Age of food allergy diagnosis, months, median (IQR)	5.0 (3.0;8.0)	6.0 (3.0;11.5)	-1.0 (-4.0;2.0)	0.513
Anaphylaxis, n (%)	1 (6.2)	2 (6.5)	1.0 (0.1;9.9)	>0.999
Asthma, n (%)	1 (6.2)	3 (9.7)	0.7 (0.1;5.7)	>0.999
Atopic dermatitis, n (%)	12 (75.0)	17 (54.8)	1.4 (0.9;2.1)	0.303
Dietary consultation, n (%)				
None	12 (75.0)	19 (61.3)	-	0.816
One time	2 (12.5)	6 (19.4)		
2-3 times	1 (6.2)	2 (6.5)		
3-4 times	1 (6.2)	1 (3.2)		
Above 4 times	0 (0.0)	3 (9.7)		
Children fed with hydrolyzed or amino-acid formula, n (%)	6 (37.5)	8 (25.8)	1.5 (0.6;3.5)	0.621
Daily intake of hydrolyzed or amino-acid formula, ml, median (IQR)	350.0 (350.0;430.0)	325.0 (175.0;400.0)	25.0 (-50.0;300.0)	0.414

Milk reintroduction (milk ladder) at timepoint of investigation n (%)				
Baked (cookie)	9 (56.2)	15 (48.4)	1.16 (0.7;2.0)	0.839
Baked (muffin)	8 (50.0)	15 (48.4)	1.03 (0.6;1.9)	>0.999
Fried (pancake)	7 (43.8)	19 (61.3)	0.71 (0.4;1.3)	0.403
Fried or baked yellow cheese	4 (25.0)	6 (19.4)	1.29 (0.4;3.9)	0.716
Hard yellow cheese	5 (31.2)	9 (29.0)	1.08 (0.4;2.7)	>0.999
Natural yogurt	4 (25.0)	12 (38.7)	0.7 (0.2;1.7)	0.539
Pasteurized milk or infant formula	4 (25.0)	7 (22.6)	1.1 (0.4;3.2)	>0.999
Raw fresh milk	2 (12.5)	3 (9.7)	1.3 (0.2;7.0)	>0.999
Age of baked milk reintroduction, months, median (IQR)	12.0 (9.0;12.0)	12.0 (8.0;12.3)	0.0 (-3.0;2.0)	0.974
Time of baked milk reintroduction – months from diagnosis, median (IQR)	6.0 (3.0;6.0)	6.0 (2.8;10.0)	0.0 (-4.0;2.0)	0.451
Settings of baked milk reintroduction, n (%)				
At home	12 (92.3)	24 (100.0)	-	0.351
At doctor's office	1 (7.7)	0 (0.0)		

IQR – interquartile range, MD – median difference (yes vs no), RR – relative risk (yes vs no), CI – confidence interval. Groups compared with Mann Whitney-U test (all numeric variables), Pearson's chi-square test (all other variables) or Fisher's exact test (duration of exclusive breastfeeding, age of complementary foods introduction, introduction of potentially allergenic foods, milk ladder: fried or baked yellow cheese, pasteurized milk or infant formula, raw fresh milk).

Table S4. Characteristics of subgroup of children with hen's eggs proteins allergy (n=30).

Variable	n (%)
Diagnostic methods	
Oral food challenge	10 (33.3)
sIgE level	18 (60.0)
Component-resolved diagnostics	5 (16.7)
Skin-prick test	3 (10.0)
Diagnosis based solely on clinical symptoms	6 (20.0)
Hen's egg reintroduction (egg ladder) at timepoint of investigation	
Baked (cookie)	13 (43.3)
Baked (muffin)	12 (40.0)
Fried (pancake, waffle)	12 (40.0)
Hard-boiled egg, well-cooked scrambled egg, french toast	6 (20.0)
Soft-boiled egg, lightly cooked egg	2 (6.7)
Kogel mogel (raw egg yolk with sugar)	0 (0.0)
Food allergy symptoms	
Skin lesions	26 (86.7)
Pruritus, including oral pruritus	11 (36.7)
Angioedema	2 (6.7)
Allergic rhinitis	6 (20.0)
Allergic conjunctivitis	2 (6.7)
Abdominal pain	11 (36.7)
Nausea, vomiting	9 (30.0)
Diarrhea	13 (43.3)
Regurgitation/spitting up	7 (23.3)
Constipation	6 (20.0)
Blood in stool	6 (20.0)
Impaired weight and/or height gain	2 (6.7)
Atopic dermatitis	16 (53.3)
Anaphylaxis (systemic reaction)	5 (16.7)
Dyspnoea	2 (6.7)

Table S5. Association between diagnosis based on oral food challenge and selected feeding practices in subgroup of children with hen's egg proteins allergy (n=30).

	**Oral Food Challenge**			
Variable	Yes	No	MD/RR (95% CI)	p
Duration of exclusive breastfeeding, n (%)				
<1 month	1 (14.3)	3 (16.7)	-	0.315
2-5 months	2 (28.6)	2 (11.1)		
6 months	4 (57.1)	7 (38.9)		
≥12 months	0 (0.0)	6 (33.3)		
Age of complementary foods introduction, n (%)				
<17 weeks	*0 (0.0)*	*0 (0.0)*	-	0.440
17-26 weeks	7 (70.0)	10 (50.0)		
>26 weeks	3 (30.0)	10 (50.0)		
Introduction of potentially allergenic foods, n (%)				
Aligned with other complementary foods	5 (50.0)	16 (80.0)	-	0.166
Delayed compared to other complementary foods	4 (40.0)	2 (10.0)		
Not introduced yet	1 (10.0)	2 (10.0)		
Declared food allergy, n (%)				
Cow's milk	6 (60.0)	14 (70.0)	0.9 (0.5;1.5)	0.690
Hen's egg	10 (100.0)	20 (100.0)	-	>0.999
Peanuts	2 (28.6)	6 (33.3)	0.9 (0.2;3.3)	>0.999
Nuts	1 (14.3)	4 (22.2)	0.6 (0.1;4.8)	>0.999
Wheat	1 (14.3)	3 (16.7)	0.9 (0.1;6.9)	>0.999
Fish	1 (14.3)	1 (5.6)	2.6 (0.2;35.7)	0.490
Seafood	0 (0.0)	0 (0.0)	-	-
Soya	1 (14.3)	3 (16.7)	0.9 (0.1;6.9)	>0.999
Healthcare specialist who diagnosed food allergy, n (%)				
Alergologist	6 (60.0)	14 (70.0)	-	0.449
Pediatrician	4 (40.0)	4 (20.0)		
Other	0 (0.0)	2 (10.0)		
Age of food allergy diagnosis, months, median (IQR)	7.5 (5.3;8.0)	7.0 (4.9;12.0)	0.5(-4.0;3.0)	0.791
Anaphylaxis, n (%)	1 (10.0)	4 (20.0)	0.5 (0.1;3.9)	0.640
Asthma, n (%)	2 (20.0)	1 (5.0)	4.0(0.4;39.0)	0.251
Atopic dermatitis, n (%)	5 (50.0)	10 (50.0)	-	>0.999

Dietary consultation, n (%)				
None	5 (50.0)	12 (60.0)		
One time	0 (0.0)	3 (15.0)		
2-3 times	3 (30.0)	2 (10.0)	-	0.547
3-4 times	1 (10.0)	1 (5.0)		
Above 4 times	1 (10.0)	2 (10.0)		
Baked hen's egg reintroduction (egg ladder) at timepoint of investigation, n (%)				
Baked (cookie)	3 (30.0)	10 (50.0)	0.6 (0.2;1.7)	0.440
Baked (muffin)	3 (30.0)	9 (45.0)	0.7 (0.2;1.9)	0.694
Fried (pancake, waffle)	2 (20.0)	10 (50.0)	0.4 (0.1;1.5)	0.235
Hard-boiled egg, well-cooked scrambled egg, French toast	0 (0.0)	6 (30.0)	-	0.074
Soft-boiled egg, lightly cooked egg	0 (0.0)	2 (10.0)	-	0.540
Kogel mogel (raw egg yolk with sugar)	0 (0.0)	0 (0.0)	-	-

IQR – interquartile range, MD – median difference (yes vs no), RR – relative risk (yes vs no), CI – confidence interval. Groups compared with Mann Whitney-U test (all numeric variables) or Fisher's exact test (all categorical variables).

Table S6. Supplement use in children with and without food allergy (n=388).

Variable	With Food Allergy (n=61)	Without Food Allergy (n=327)	*p*
Vitamin D, n (%)			
Daily	51 (83.6)	247 (75.5)	
Irregularly	8 (13.1)	62 (19.0)	0.386
Not used	2 (3.3)	18 (5.5)	
DHA, n (%)			
Daily	12 (19.7)	71 (21.7)	
Irregularly	10 (16.4)	45 (13.8)	0.838
Not used	39 (63.9)	211 (64.5)	
Probiotic, n (%)			
Daily	14 (23.0)	52 (15.9)	
Irregularly	14 (23.0)	68 (20.8)	0.314
Not used	33 (54.1)	207 (63.3)	
Iron, n (%)			
Daily	4 (6.6)	20 (6.1)	
Irregularly	4 (6.6)	18 (5.5)	0.848
Not used	53 (86.9)	289 (88.4)	
Calcium, n (%)			
Daily	1 (1.6)	10 (3.1)	
Irregularly	2 (3.3)	20 (6.1)	0.726
Not used	58 (95.1)	297 (90.8)	
Multivitamin and mineral supplements, n (%)			
Daily	4 (6.6)	31 (9.5)	
Irregularly	4 (6.6)	24 (7.3)	0.735
Not used	53 (86.9)	272 (83.2)	
Other, n (%)			
Daily	5 (8.2)	28 (8.6)	
Irregularly	2 (3.3)	20 (6.1)	0.670
Not used	54 (88.5)	279 (85.3)	

Groups compared with Pearson's chi-square test (all other variables) or Fisher's exact test (iron, calcium).

Table S7. Association between any breastfeeding duration and diet and specific food group diversity in children with food allergy (n=61).

	Duration of breastfeeding					
**Variable**	**<1 month**	**1.5-5 months**	**6 months**	**6-12 months**	**>12 months**	**p**
Children with feeding difficulties, n (%)	3 (30.0)	3 (30.0)	1 (25.0)	1 (5.3)	2 (12.5)	0.217
Food allergen diversity [0-8], median (IQR)	4.0 (4.0;5.0)[k]	6.0 (2.8;6.8)	4.5 (3.0;6.0)	7.0 (5.0;8.0)[k]	6.5 (5.0;8.0)	**0.014**
The WHO Minimum Dietary Diversity [0-8], median (IQR)	6.0 (6.0;7.0)	6.5 (6.0;7.0)	7.0 (6.8;7.0)	7.0 (6.0;7.0)	7.0 (6.0;7.0)	0.539
Fruit and vegetable diversity [0-5], median (IQR)	5.0 (4.3;5.0)	5.0 (5.0;5.0)	4.5 (4.0;5.0)	5.0 (4.5;5.0)	5.0 (4.8;5.0)	0.858
Overall diet diversity [0-15], mean±SD	11.7±3.40	11.4±2.72	12.25±1.0	12.8±1.6	12.3±1.8	0.536
Food groups diversity [0-11], median (IQR)	10.0 (8.3;10.0)	10.0 (8.5;10.8)	10.5 (9.8;11.0)	10.0 (10.0;11.0)	10.0 (9.0;10.0)	0.568
Foods diversity (43 products) [0-43], median (IQR)	32.0 (29.5;36.8)	31.0 (24.0;32.8)	27.0 (26.0;30.0)	33.0 (30.0;36.5)	30.0 (28.8;34.3)	0.550

SD – standard deviation, IQR – interquartile range. Groups compared with ANOVA (FFQ – potentially allergenic food in total group, FFQ – overall diet diversity in allergy group) or Kruskal-Wallis test (all other comparisons).

* Data available for n=61 in allergy group

Table S8. Association between exclusive breastfeeding duration and diet and specific food group diversity in children with food allergy (n=61).

	Duration of exclusive breastfeeding				
**Variable**	**<1 month**	**2-5 months**	**6 months**	**≥7 months**	**p**
Children with feeding difficulties, n (%)	3 (25.0)	2 (18.2)	1 (4.8)	1 (11.1)	0.096
Food allergen diversity [0-8], median (IQR)	5.5 (4.0;7.0)	6.0 (5.5;7.0)	6.0 (5.0;8.0)	7.0 (6.0;8.0)	0.685
The WHO Minimum Dietary Diversity [0-8], median (IQR)	7.0 (6.0;7.0)	6.0 (5.5;7.0)	7.0 (6.0;7.0)	7.0 (7.0;7.0)	0.402
Fruit and vegetable diversity [0-5], median (IQR)	5.0 (5.0;5.0)	5.0 (5.0;5.0)	5.0 (5.0;5.0)	5.0 (4.0;5.0)	0.317
Overall diet diversity [0-15], mean±SD	13.3±1.4	11.8±2.3	12.4±1.7	12.6±1.7	0.230
Food groups diversity [0-11], median (IQR)	10.5 (10.0;11.0)	10.0 (8.0;11.0)	10.0 (10.0;10.0)	10.0 (9.0;11.0)	0.537
Foods diversity (43 products) [0-43], median (IQR)	35.0 (31.0;37.0)	32.0 (24.0;34.5)	30.0 (28.0;36.0)	32.0 (30.0;33.0)	0.296

SD – standard deviation, IQR – interquartile range. Groups compared with ANOVA (MCH-FS Feeding Difficulties in total group and in allergy group, FFQ – overall diet diversity in allergy group) or Kruskal-Wallis test (all other comparisons).

Table S9. Association between feeding difficulties, diet and specific food groups diversity and feeding with human milk substitutes in children with food allergy (n=61).

	Fed with human milk substitutes			
**Variable**	**Yes**	**No**	**MD (95% CI)**	**p**
Children with feeding difficulties, n (%)	7 (30.4)	4 (10.5)	2.9 (1.0;8.8)	0.084
Food allergen diversity [0-8], mean±SD	5.1±2.3	5.9±1.7	-0.8 (-1.8;0.2)	0.133
The WHO Minimum Dietary Diversity [0-8], median (IQR)	7.0 (6.0;7.0)	7.0 (6.0;7.0)	0.0 (0.0;0.0)	0.637
Fruit and vegetable diversity [0-5], median (IQR)	5.0 (5.0;5.0)	5.0 (4.0;5.0)	0.0 (0.0;0.0)	0.257
Overall diet diversity [0-15], median (IQR)	13.0 (12.0;14.0)	12.5 (11.0;13.8)	0.5 (-1.0;1.0)	0.717
Food groups diversity [0-11], median (IQR)	10.0 (9.0;11.0)	10.0 (9.3;10.8)	0.0 (-1.0;0.0)	0.943
Foods diversity (43 products) [0-43], median (IQR)	32.0 (27.0;37.0)	32.0 (28.3;34.0)	0.0 (-2.0;4.0)	0.687

SD – standard deviation, IQR – interquartile range, MD – mean or median difference (yes vs no), CI – confidence interval. Groups compared with t Student test (FFQ – Food allergen diversity in allergy group), or Mann-Whitney U test (all other comparisons).

Table S10. Association between feeding difficulties, diet and specific food groups diversity and consuming plant-based beverages in children with food allergy (n=61).

	Consuming plant-based beverages			
**Variable**	**Yes**	**No**	**MD (95% CI)**	**p**
Children with feeding difficulties, n (%)	7 (24.1)	4 (12.5)	1.9 (0.6;5.9)	0.397
Food allergen diversity [0-8], mean±SD	5.8±1.7	5.4±2.2	0.4 (-0.6;1.4)	0.410
The WHO Minimum Dietary Diversity [0-8], median (IQR)	7.0 (6.0;7.0)	7.0 (6.0;7.0)	0.0 (0.0;0.0)	0.740
Fruit and vegetable diversity [0-5], median (IQR)	5.0 (4.0;5.0)	5.0 (4.8;5.0)	0.0 (0.0;0.0)	0.895
Overall diet diversity [0-15], median (IQR)	12.0 (11.0;13.0)	13.0 (11.0;14.0)	-1.0 (-2.0;0.0)	0.176
Food groups diversity [0-11], median (IQR)	10.0 (10.0;10.0)	10.0 (9.0;11.0)	0.0 (0.0;1.0)	0.725
Foods diversity (43 products) [0-43], median (IQR)	31.0 (29.0;33.0)	32.50(27.8;37.0)	-1.5 (-4.0;2.0)	0.381

SD – standard deviation, IQR – interquartile range, MD – mean or median difference (yes vs no), CI – confidence interval. Groups compared with t Student test (FFQ – Food allergen diversity), t Welch test or Mann-Whitney U test (all other comparisons).

Table S11. Association between declared food allergy symptoms and delay in complementary foods introduction in children with food allergy (n=61).

Variable	Age of complementary foods introduction		RR (95% CI)	p
	>26 weeks	≤26 weeks		
Skin lesions, n (%)	18 (90.0)	36 (87.8)	1.0 (0.9;1.2)	>0.999
Pruritus, including oral pruritus, n (%)	8 (40.0)	6 (14.6)	2.7 (1.1;6.8)	0.059
Angioedema, n (%)	2 (10.0)	0 (0.0)	-	0.104
Allergic rhinitis, n (%)	5 (25.0)	9 (22.0)	1.1 (0.4;3.0)	>0.999
Allergic conjunctivitis, n (%)	1 (5.0)	3 (7.3)	0.7 (0.1;6.2)	>0.999
Abdominal pain, n (%)	8 (40.0)	13 (31.7)	1.3 (0.6;2.5)	0.724
Nausea, vomiting, n (%)	4 (20.0)	9 (22.0)	0.9 (0.3;2.6)	>0.999
Diarrhea, n (%)	5 (25.0)	13 (31.7)	0.8 (0.3;1.9)	0.810
Regurgitation/spitting up, n (%)	5 (25.0)	7 (17.1)	1.5 (0.5;4.0)	0.505
Constipation, n (%)	3 (15.0)	10 (24.4)	0.6 (0.2;2.0)	0.516
Blood in stool, n (%)	4 (20.0)	7 (17.1)	1.2 (0.4;3.5)	>0.999
Impaired weight and/or height gain, n (%)	1 (5.0)	6 (14.6)	0.3 (0.0;2.7)	0.409
Atopic dermatitis, n (%)	13 (65.0)	18 (43.9)	1.5 (0.9;2.4)	0.202
Anaphylaxis (systemic reaction), n (%)	2 (10.0)	3 (7.3)	1.4 (0.3;7.5)	>0.999
Dyspnoea, n (%)	1 (5.0)	3 (7.3)	0.7 (0.1;6.2)	>0.999

RR – relative risk (>26 weeks vs ≤26 weeks), CI – confidence interval. Groups compared with Pearson's chi-square test (itching, swelling of the oral cavity; allergic rhinitis, abdominal pain, diarrhea, atopic dermatitis) or Fisher's exact test (all other variables).

Table S12. Association between declared food allergy symptoms and delay in introduction of potentially allergenic foods in children with food allergy (n=61).

	**Introduction of potentially allergenic complementary food compared to other complementary foods**			
**Variable**	**Delayed**	**Not delayed**	**RR (95% CI)**	**p**
Skin lesions, n (%)	35 (85.4)	16 (94.1)	0.9 (0.8;1.1)	0.661
Pruritus, including oral pruritus, n (%)	9 (22.0)	3 (17.6)	1.2 (0.4;4.0)	>0.999
Angioedema, n (%)	2 (4.9)	0 (0.0)	-	>0.999
Allergic rhinitis, n (%)	8 (19.5)	6 (35.3)	0.6 (0.2;1.3)	0.311
Allergic conjunctivitis, n (%)	3 (7.3)	1 (5.9)	1.2 (0.1;11.1)	>0.999
Abdominal pain, n (%)	11 (26.8)	8 (47.1)	0.6 (0.3;1.2.0)	0.235
Nausea, vomiting, n (%)	9 (22.0)	4 (23.5)	0.9 (0.3;2.6)	>0.999
Diarrhea, n (%)	10 (24.4)	6 (35.3)	0.7 (0.3;1.6)	0.601
Regurgitation/spitting up, n (%)	6 (14.6)	4 (23.5)	0.6 (0.2;1.9)	0.458
Constipation, n (%)	8 (19.5)	3 (17.6)	1.1 (0.3;3.7)	>0.999
Blood in stool, n (%)	7 (17.1)	4 (23.5)	0.7 (0.2;2.2)	0.715
Impaired weight and/or height gain, n (%)	6 (14.6)	1 (5.9)	2.5 (0.3;19.1)	0.661
Atopic dermatitis, n (%)	22 (53.7)	7 (41.2)	1.3 (0.7;2.5)	0.564
Anaphylaxis (systemic reaction), n (%)	4 (9.8)	0 (0.0)	-	0.310
Dyspnoea, n (%)	3 (7.3)	1 (5.9)	1.2 (0.1;11.1)	>0.999

Children who were not introduced to Food allergen diversity yet were excluded. RR – relative risk (delayed vs not delayed), CI – confidence interval. Groups compared with Pearson's chi-square test (abdominal pain, diarrhea, atopic dermatitis) or Fisher's exact test (all other variables).
